# Supplementary material for: Trends and outcomes of non-primary PCI at sites without cardiac surgery on-site: The early Michigan experience
Source: PLoS One. 2020 Aug 26;15(8):e0238048. doi: 10.1371/journal.pone.0238048 (PMC7449474; doi:10.1371/journal.pone.0238048)
Supplement: S1 Table — (DOCX) [file pone.0238048.s001.docx]

**S1 Table**: **Baseline variables included in the propensity-matched model**

| **Characteristic** |
| --- |
| Age |
| Weight |
| Height |
| Diabetes and diabetes control (diet, oral medication, insulin) |
| Chronic lung disease |
| Peripheral Arterial Disease |
| Acute Congestive Heart Failure within 2 weeks prior to PCI |
| NYHA class within 2 weeks |
| Pre-PCI Left Ventricular Ejection Fraction |
| PCI status (Elective, Urgent, Emergent, Salvage) |
| CAD Presentation (STEMI, NSTEMI, Unstable Angina, Stable Angina, other) |
| PCI indication (primary PCI, PCI for other STEMI, PCI after initial thrombolytic therapy, etc). |
| Cardiac arrest |
| Anginal Class |
| Cardiogenic shock |
| Pre-procedural vasopressor use |
| Atrial fibrillation |
| **Pre-procedural labs** |
| Hemoglobin |
| CK-MB |
| Troponin |
| Creatinine |

*CAD = Coronary Artery Disease; NYHA = New York Heart Association; NSTEMI = non-ST elevation Myocardial Infarction; STEMI = ST-elevation Myocardial Infarction; PCI = Percutaneous Coronary Intervention; CK-MB = Creatinine Kinase-Muscle/Brain*
