# Supplementary material for: Trends and outcomes of non-primary PCI at sites without cardiac surgery on-site: The early Michigan experience
Source: PLoS One. 2020 Aug 26;15(8):e0238048. doi: 10.1371/journal.pone.0238048 (PMC7449474; doi:10.1371/journal.pone.0238048)
Supplement: S2 Table — (DOCX) [file pone.0238048.s002.docx]

**S2 Table: Baseline characteristics of overall unmatched non-primary PCI cohort during study period**

|  | **Sites with Surgery** | **%cases** | **Sites Without Surgery** | **%cases** | **P-value** | **ASD (%)** |
| --- | --- | --- | --- | --- | --- | --- |
| N | 46,096 |  | 4,721 |  |  |  |
| *Demographics* |  |  |  |  |  |  |
| Age, yrs | 66.7 ± 11.5 |  | 64.9 ± 11.4 |  | p < 0.001 | 15.38 |
| Male | 30,925 | 67.1% | 3,086 | 65.4% | p = 0.017 | 3.64 |
| White | 39,747 | 86.2% | 4,139 | 87.7% | p = 0.006 | 4.29 |
| *Clinical History* |  |  |  |  |  |  |
| Hypertension | 41,024 | 89.0% | 4,031 | 85.4% | p < 0.001 | 10.83 |
| Dyslipidemia | 38,825 | 84.3% | 3,652 | 77.4% | p < 0.001 | 17.57 |
| Diabetes Mellitus | 19,753 | 42.9% | 1,934 | 41.0% | p = 0.015 | 3.72 |
| Current/Recent Smoker (<1 year) | 11,182 | 24.3% | 1,319 | 37.9% | p < 0.001 | 8.38 |
| Family History of Premature CAD | 5,227 | 11.3% | 689 | 14.6% | p < 0.001 | 9.70 |
| Peripheral Arterial Disease | 7,580 | 16.4% | 539 | 11.4% | p < 0.001 | 14.55 |
| Prior Myocardial Infarction | 17,406 | 37.8% | 1,579 | 33.5% | p < 0.001 | 9.02 |
| Prior PCI | 23,667 | 51.4% | 2,068 | 43.9% | p < 0.001 | 15.02 |
| Prior CABG | 9,222 | 20.0% | 526 | 11.1% | p < 0.001 | 24.62 |
| Prior Heart Failure | 10,109 | 21.9% | 770 | 16.3% | p < 0.001 | 14.33 |
| Heart Failure within 2 weeks | 6,924 | 15.0% | 627 | 13.3% | p = 0.002 | 4.86 |
| Prior Valve Surgery/Procedure | 1,110 | 2.4% | 82 | 1.7% | p = 0.004 | 4.70 |
| Chronic Lung Disease | 9,523 | 20.7% | 820 | 17.4% | p < 0.001 | 8.39 |
| Currently on Dialysis | 1,429 | 3.1% | 135 | 2.9% | p = 0.359 | 1.42 |
| Cerebrovascular Disease | 7,898 | 17.1% | 594 | 12.6% | p < 0.001 | 12.82 |
| GFR, mL/min/1.73m^2^ (CKD-EPI) | 72.3 ± 24.4 |  | 75.5 ± 24.1 |  | p < 0.001 | 13.28 |
| Body Mass Index, kg/m^2^ | 30.9 ± 7.9 |  | 30.9 ± 6.7 |  | p = 0.922 | 0.14 |
| *CAD Presentation* |  |  |  |  |  |  |
| NSTEMI | 13,076 | 28.4% | 1,638 | 34.7% | p < 0.001 | 13.65 |
| Unstable Angina | 22,988 | 49.9% | 2,170 | 46.0% | p < 0.001 | 7.82 |
| Stable Angina | 6,286 | 13.6% | 559 | 11.8% | p < 0.001 | 5.39 |
| Symptoms unlikely to be ischemic | 1,675 | 3.6% | 160 | 3.4% | p = 0.391 | 1.33 |
| No symptoms, no angina | 2,071 | 4.5% | 194 | 4.1% | p = 0.224 | 1.89 |
| *Access Site* |  |  |  |  |  |  |
| Femoral | 25,115 | 54.5% | 2,019 | 42.8% | p < 0.001 | 23.58 |
| Radial | 20,840 | 45.2% | 2,684 | 56.9% | p < 0.001 | 23.49 |
| Brachial | 73 | 0.2% | 8 | 0.2% | p = 0.855 | 0.28 |
| Other | 65 | 0.1% | 8 | 0.2% | p = 0.622 | 0.72 |
| *Peri-Procedural Variables & Complications* |  |  |  |  |  |  |
| IABP | 316 | 0.7% | 29 | 0.6% | p = 0.572 | 0.88 |
| Perforation | 204 | 0.4% | 16 | 0.3% | p = 0.303 | 1.66 |
| Significant Dissection | 358 | 0.8% | 27 | 0.6% | p = 0.123 | 2.50 |
| Pre-PCI LVEF, mean % + SD | 52.4 ± 12.9 |  | 52.9 ± 12.0 |  | p = 0.020 | 3.90 |
| Contrast Volume, mean mL + SD | 158.7 ± 66.4 |  | 161.2 ± 67.1 |  | p = 0.012 | 3.86 |
| *Vessel(s) Intervened Upon* |  |  |  |  |  |  |
| All Left Main | 1,826 | 4.0% | 49 | 1.0% | p < 0.001 | 18.81 |
| Left Anterior Descending | 19,861 | 43.1% | 2,056 | 43.6% | p = 0.547 | 0.94 |
| Left Circumflex | 13,810 | 30.0% | 1,275 | 27.0% | p < 0.001 | 6.54 |
| Right Coronary Artery | 15,144 | 32.9% | 1,569 | 33.2% | p = 0.603 | 0.81 |
| Bypass Graft | 2,957 | 6.4% | 163 | 3.5% | p < 0.001 | 13.71 |
| Chronic Total Occlusion | 2,220 | 4.8% | 89 | 1.9% | p < 0.001 | 16.34 |
| Bifurcation | 3,565 | 7.7% | 378 | 8.0% | p = 0.511 | 1.01 |
| Lesion Data Missing | 150 | 0.3% | 109 | 2.3% | p < 0.001 | 17.46 |
| *Device Used* |  |  |  |  |  |  |
| Bare Metal Stent only | 2,261 | 4.9% | 252 | 5.3% | p = 0.277 | 1.96 |
| Drug-Eluting Stent only | 38,980 | 84.6% | 4096 | 86.8% | p < 0.001 | 6.28 |
| Balloon only | 3,525 | 7.6% | 275 | 5.8% | p < 0.001 | 7.27 |
| BMS + DES | 185 | 0.4% | 8 | 0.2% | p = 0.002 | 4.35 |
| Device Data Missing | 1,145 | 2.5% | 90 | 1.9% | p < 0.001 | 3.94 |
| Atherectomy device | 1,606 | 3.5% | 0 | 0.0% | p < 0.001 | 26.87 |
| *Intra-procedural Medications* |  |  |  |  |  |  |
| Intravenous UFH | 42,223 | 91.8% | 4,561 | 96.8% | p < 0.001 | 21.80 |
| Bivalirudin | 7,621 | 16.5% | 446 | 9.5% | p < 0.001 | 21.18 |
| Bivalirudin + GPI | 220 | 0.5% | 23 | 0.5% | p = 1.000 | 0.14 |
| GPI + UFH | 6,953 | 15.1% | 1,118 | 23.7% | p < 0.001 | 21.88 |
| *Oral Antiplatelets Used* |  |  |  |  |  |  |
| Aspirin | 45,415 | 99.3% | 4,323 | 92.7% | p < 0.001 | 33.90 |
| Clopidogrel | 25,771 | 56.1% | 2,164 | 46.1% | p < 0.001 | 20.27 |
| Prasugrel | 4,149 | 9.0% | 220 | 4.7% | p < 0.001 | 17.26 |
| Ticagrelor | 15,462 | 33.6% | 2,048 | 43.5% | p < 0.001 | 20.40 |

*ASD = absolute standardized difference; BMS = bare metal stent; CAD = coronary artery disease; CABG = coronary artery bypass graft; CKD-EPI = Chronic Kidney Disease Epidemiology Collaboration; DES = drug eluting stent; GFR = glomerular filtration rate; GPI = glycoprotein inhibitor; IABP = intra-aortic balloon pump; NSTEMI = non-ST elevation myocardial infarction; UFH = unfractionated heparin*
