# Supplementary material for: Trends and outcomes of non-primary PCI at sites without cardiac surgery on-site: The early Michigan experience
Source: PLoS One. 2020 Aug 26;15(8):e0238048. doi: 10.1371/journal.pone.0238048 (PMC7449474; doi:10.1371/journal.pone.0238048)
Supplement: S3 Table — (DOCX) [file pone.0238048.s003.docx]

**S3 Table: Overall clinical and procedural outcomes, and major complications of unmatched non-primary PCI cohorts at sites with and without on-site surgery**

|  | **Sites with Surgery** | **%cases** | **Sites Without Surgery** | **%cases** | **P-value** | **ASD (%)** |
| --- | --- | --- | --- | --- | --- | --- |
| *N* | 46,096 |  | 4,721 |  |  |  |
| In-Hospital Mortality | 240 | 0.5% | 21 | 0.4% | p = 0.487 | 1.09 |
| Major Bleeding | 194/36,963 | 0.5% | 14/3,310 | 0.4% | p = 0.526 | 1.48 |
| RBC/Whole Blood Transfusion | 784 | 1.7% | 54 | 1.1% | p = 0.004 | 4.71 |
| Other Vascular Complications Requiring Transfusion | 137 | 0.3% | 7 | 0.1% | p = 0.067 | 3.16 |
| CVA/Stroke | 125 | 0.3% | 6 | 0.1% | p = 0.063 | 3.23 |
| Cardiogenic Shock | 376 | 0.8% | 41 | 0.9% | p = 0.699 | 0.58 |
| Heart Failure | 860 | 1.9% | 49 | 1.0% | p < 0.001 | 6.92 |
| Subacute stent thrombosis | 56 | 0.1% | 6 | 0.1% | p = 0.827 | 0.16 |
| Target lesion revascularization | 189 | 0.4% | 9 | 0.2% | p = 0.019 | 4.01 |
| CABG (urgent/emergent status) | 222 | 0.5% | 15 | 0.3% | p = 0.116 | 2.59 |
| Contrast-Induced Nephropathy | 1,603/35,596 | 4.0% | 120/3,470 | 3.5% | p = 0.095 | 3.11 |
| New Requirement for Dialysis | 84 | 0.2% | 5 | 0.1% | p = 0.276 | 2.01 |
| Length of Stay (days) | 2.8 ± 12.1 |  | 2.6 ± 3.0 |  | p = 0.001 | 2.63 |

*ASD = absolute standardized difference; CABG = coronary artery bypass graft; CVA = cerebrovascular accident*
