# Supplementary material for: Trends and outcomes of non-primary PCI at sites without cardiac surgery on-site: The early Michigan experience
Source: PLoS One. 2020 Aug 26;15(8):e0238048. doi: 10.1371/journal.pone.0238048 (PMC7449474; doi:10.1371/journal.pone.0238048)
Supplement: S4 Table — (DOCX) [file pone.0238048.s004.docx]

**S4 Table: Clinical and procedural outcomes, and major complications at sites with and without on-site surgery excluding high-risk patients**

|  | **Sites with Surgery** | **%cases** | **Sites Without Surgery** | **%cases** | **P-value** | **ASD (%)** |
| --- | --- | --- | --- | --- | --- | --- |
| *N* | 3,967 |  | 3,967 |  |  |  |
| Primary Composite Endpoint | 116 | 2.9% | 146 | 3.7% | p = 0.068 | 4.23 |
| In-Hospital Mortality | 10 | 0.3% | 15 | 0.4% | p = 0.317 | 2.25 |
| Major Bleeding | 9 | 0.3% | 13 | 0.5% | p = 0.286 | 3.07 |
| RBC/Whole Blood Transfusion | 37 | 0.9% | 45 | 1.1% | p = 0.374 | 1.99 |
| Other Vascular Complications Requiring Transfusion | 8 | 0.2% | 7 | 0.2% | p = 0.797 | 0.58 |
| CVA/Stroke | 11 | 0.3% | 5 | 0.1% | p = 0.134 | 3.37 |
| Cardiogenic Shock | 14 | 0.4% | 29 | 0.7% | p = 0.022 | 5.16 |
| Heart Failure | 50 | 1.3% | 30 | 0.8% | p = 0.025 | 5.04 |
| Subacute stent thrombosis | 4 | 0.1% | 6 | 0.2% | p = 0.754 | 1.42 |
| Target lesion revascularization | 9 | 0.2% | 7 | 0.2% | p = 0.803 | 1.12 |
| CABG (urgent/emergent status) | 15 | 0.4% | 11 | 0.3% | p = 0.433 | 1.76 |
| Contrast-Induced Nephropathy | 49 | 1.5% | 51 | 1.8% | p = 0.313 | 2.65 |
| New Requirement for Dialysis | 4 | 0.1% | 4 | 0.1% | p = 0.999 | 0.00 |
| Length of Stay (days) | 2.5 ± 2.9 |  | 2.5 ± 2.9 |  | p = 0.643 | 1.04 |

*ASD = absolute standardized difference; CABG = coronary artery bypass graft; CVA = cerebrovascular accident; MACE = major adverse cardiovascular event; PCI = percutaneous coronary intervention; RBC = red blood cell*
