# Supplementary material for: Trends and outcomes of non-primary PCI at sites without cardiac surgery on-site: The early Michigan experience
Source: PLoS One. 2020 Aug 26;15(8):e0238048. doi: 10.1371/journal.pone.0238048 (PMC7449474; doi:10.1371/journal.pone.0238048)
Supplement: S5 Table — (DOCX) [file pone.0238048.s005.docx]

**S5 Table: Clinical and procedural outcomes, and major complications at sites with and without on-site surgery of high-risk patient subset**

|  | **Sites with Surgery** | **%cases** | **Sites Without Surgery** | **%cases** | **P-value** | **ASD (%)** |
| --- | --- | --- | --- | --- | --- | --- |
| *N* | 651 |  | 651 |  |  |  |
| Primary Composite Endpoint | 41 | 6.3% | 32 | 4.9% | p = 0.335 | 6.01 |
| In-Hospital Mortality | 4 | 0.6% | 6 | 0.9% | p = 0.753 | 3.52 |
| Major Bleeding | 1 | 0.2% | 1 | 0.2% | p = 1.000 | 0.46 |
| RBC/Whole Blood Transfusion | 14 | 2.2% | 8 | 1.2% | p = 0.282 | 7.14 |
| Other Vascular Complications Requiring Transfusion | 2 | 0.3% | 0 | 0.0% | p = 0.500 | 7.85 |
| CVA/Stroke | 1 | 0.2% | 1 | 0.2% | p = 1.000 | 0.01 |
| Cardiogenic Shock | 5 | 0.8% | 12 | 1.8% | p = 0.094 | 9.5 |
| Heart Failure | 26 | 4.0% | 19 | 2.9% | p = 0.363 | 5.86 |
| Subacute stent thrombosis | 2 | 0.3% | 0 | 0.0% | p = 0.500 | 7.85 |
| Target lesion revascularization | 4 | 0.6% | 1 | 0.2% | p = 0.374 | 7.46 |
| CABG (urgent/emergent status) | 6 | 0.9% | 4 | 0.6% | p = 0.753 | 3.52 |
| Contrast-Induced Nephropathy | 15 | 2.6% | 13 | 2.7% | p = 1.000 | 0.32 |
| New Requirement for Dialysis | 0 | 0.0% | 1 | 0.2% | p = 0.500 | 5.55 |
| Length of Stay (days) | 3.5 ± 4.1 |  | 3.1 ± 3.1 |  | p = 0.074 | 9.92 |

*ASD = absolute standardized difference; CABG = coronary artery bypass graft; CVA = cerebrovascular accident; MACE = major adverse cardiovascular event; PCI = percutaneous coronary intervention; RBC = red blood cell*
