# Supplementary material for: Trends and outcomes of non-primary PCI at sites without cardiac surgery on-site: The early Michigan experience
Source: PLoS One. 2020 Aug 26;15(8):e0238048. doi: 10.1371/journal.pone.0238048 (PMC7449474; doi:10.1371/journal.pone.0238048)
Supplement: S6 Table — (DOCX) [file pone.0238048.s006.docx]

**S6 Table: Baseline characteristics of propensity-matched cohorts of non-primary PCI’s not including high-risk cases at sites with and without cardiac surgery**

|  | **Sites with Surgery** | **%cases** | **Sites Without Surgery** | **%cases** | **P-value** | **ASD (%)** |
| --- | --- | --- | --- | --- | --- | --- |
| N | 3,967 |  | 3,967 |  |  |  |
| *Demographics* |  |  |  |  |  |  |
| Age, yrs | 65.1 ± 11.9 |  | 64.9 ± 11.5 |  | p = 0.592 | 1.20 |
| Male | 2,674 | 67.4% | 2,565 | 64.7% | p = 0.010 | 5.80 |
| White | 3,637 | 84.9% | 3,466 | 87.4% | p = 0.001 | 7.22 |
| *Clinical History* |  |  |  |  |  |  |
| Hypertension | 3,461 | 87.3% | 3,372 | 85.0% | p = 0.004 | 6.56 |
| Dyslipidemia | 3,226 | 81.4% | 3,069 | 77.4% | p < 0.001 | 9.95 |
| Diabetes Mellitus | 1,647 | 41.5% | 1,608 | 40.5% | p = 0.363 | 2.04 |
| Current/Recent Smoker (<1 year) | 1,035 | 26.1% | 1,101 | 27.8% | p = 0.096 | 3.74 |
| Family History of Premature CAD | 460 | 11.6% | 582 | 14.7% | p < 0.001 | 9.11 |
| Peripheral Arterial Disease | 455 | 11.5% | 445 | 11.2% | p = 0.717 | 0.81 |
| Prior Myocardial Infarction | 1,379 | 34.8% | 1,304 | 32.9% | p = 0.075 | 4.00 |
| Prior PCI | 1,903 | 48.0% | 1,722 | 43.5% | p < 0.001 | 9.08 |
| Prior CABG | 643 | 16.2% | 411 | 10.4% | p < 0.001 | 17.28 |
| Prior Heart Failure | 648 | 16.3% | 598 | 15.1% | p = 0.120 | 3.49 |
| Heart Failure within 2 weeks | 433 | 10.9% | 469 | 11.8% | p = 0.196 | 2.91 |
| Prior Valve Surgery/Procedure | 67 | 1.7% | 64 | 1.6% | p = 0.794 | 0.59 |
| Chronic Lung Disease | 740 | 18.7% | 680 | 17.1% | p = 0.077 | 3.97 |
| Currently on Dialysis | 110 | 2.8% | 110 | 2.8% | p = 0.997 | 0.01 |
| Cerebrovascular Disease | 597 | 15.1% | 520 | 13.1% | p = 0.013 | 5.60 |
| GFR, mL/min/1.73m^2^ (CKD-EPI) | 74.9 ± 24.4 |  | 75.5 ± 24.0 |  | p = 0.261 | 2.53 |
| Body Mass Index, kg/m^2^ | 31.2 ± 8.3 |  | 31.0 ± 6.8 |  | p = 0.419 | 1.82 |
| *CAD Presentation* |  |  |  |  |  |  |
| NSTEMI | 1,382 | 34.80% | 1,380 | 34.8% | p = 0.962 | 0.11 |
| Unstable Angina | 1,798 | 45.30% | 1,806 | 45.5% | p = 0.857 | 0.41 |
| Stable Angina | 488 | 12.30% | 495 | 12.5% | p = 0.811 | 0.54 |
| Symptoms unlikely to be ischemic | 137 | 3.50% | 126 | 3.2% | p = 0.490 | 1.55 |
| No symptoms, no angina | 162 | 4.10% | 160 | 4.0% | p = 0.909 | 0.26 |
| *Access Site* |  |  |  |  |  |  |
| Femoral | 1,960 | 49.40% | 1,690 | 42.6% | p < 0.001 | 13.64 |
| Radial | 1,992 | 50.20% | 2,263 | 57.1% | p < 0.001 | 13.79 |
| Brachial | 8 | 0.20% | 5 | 0.1% | p = 0.406 | 1.87 |
| Other | 7 | 0.20% | 7 | 0.2% | p = 0.999 | 0.00 |
| *Peri-Procedural Variables & Complications* |  |  |  |  |  |  |
| IABP | 17 | 0.40% | 20 | 0.5% | p = 0.620 | 1.11 |
| Perforation | 8 | 0.20% | 12 | 0.3% | p = 0.369 | 2.02 |
| Significant Dissection | 22 | 0.60% | 23 | 0.6% | p = 0.878 | 0.34 |
| Pre-PCI LVEF, mean % + SD | 54.7 ± 9.9 |  | 54.7 ± 9.8 |  | p = 0.803 | 0.64 |
| Contrast Volume, mean mL + SD | 151.2 ± 60.5 |  | 159.2 ± 66.4 |  | p < 0.001 | 12.57 |

*ASD = absolute standardized difference; BMS = bare metal stent; CAD = coronary artery disease; CABG = coronary artery bypass graft; CKD-EPI = Chronic Kidney Disease Epidemiology Collaboration; DES = drug eluting stent; GFR = glomerular filtration rate; GPI = glycoprotein inhibitor; IABP = intra-aortic balloon pump; NSTEMI = non-ST elevation myocardial infarction; UFH = unfractionated heparin*
