# Supplementary figures and images for: Trends and outcomes of non-primary PCI at sites without cardiac surgery on-site: The early Michigan experience
Source: PLoS One. 2020 Aug 26;15(8):e0238048. doi: 10.1371/journal.pone.0238048 (PMC7449474; doi:10.1371/journal.pone.0238048)

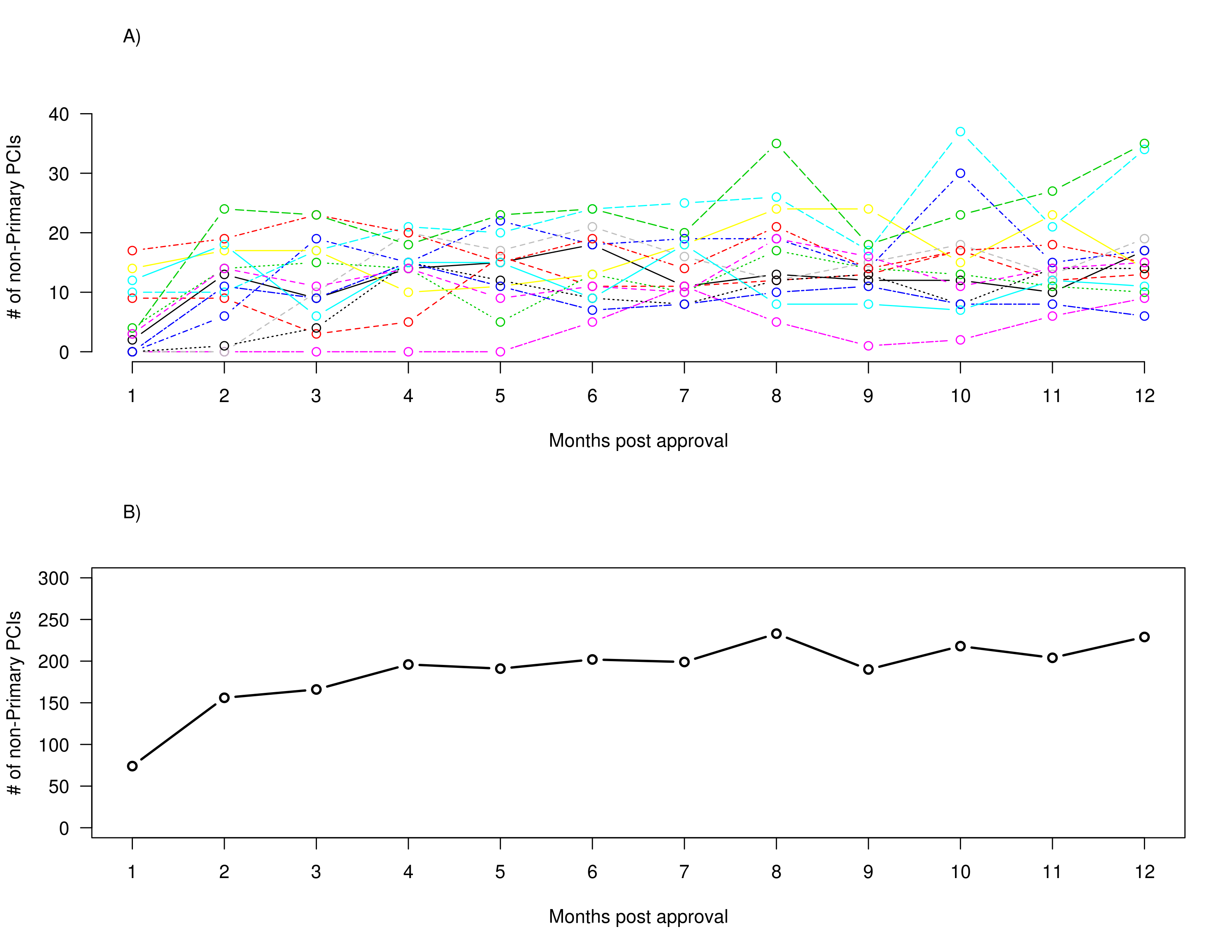

Supplement: S1 Fig — Non-primary PCI volume from time of approval through first 12-month period post-approval for (a) all 14 participating sites individually, and (b) combined PCI volume for all sites during each of the months. Notable heterogeneity seen between sites, with progressive ramp-up of volume during the first 12 month period for each site post-initiation of non-primary PCI practices. (TIF) [file pone.0238048.s007.tif]

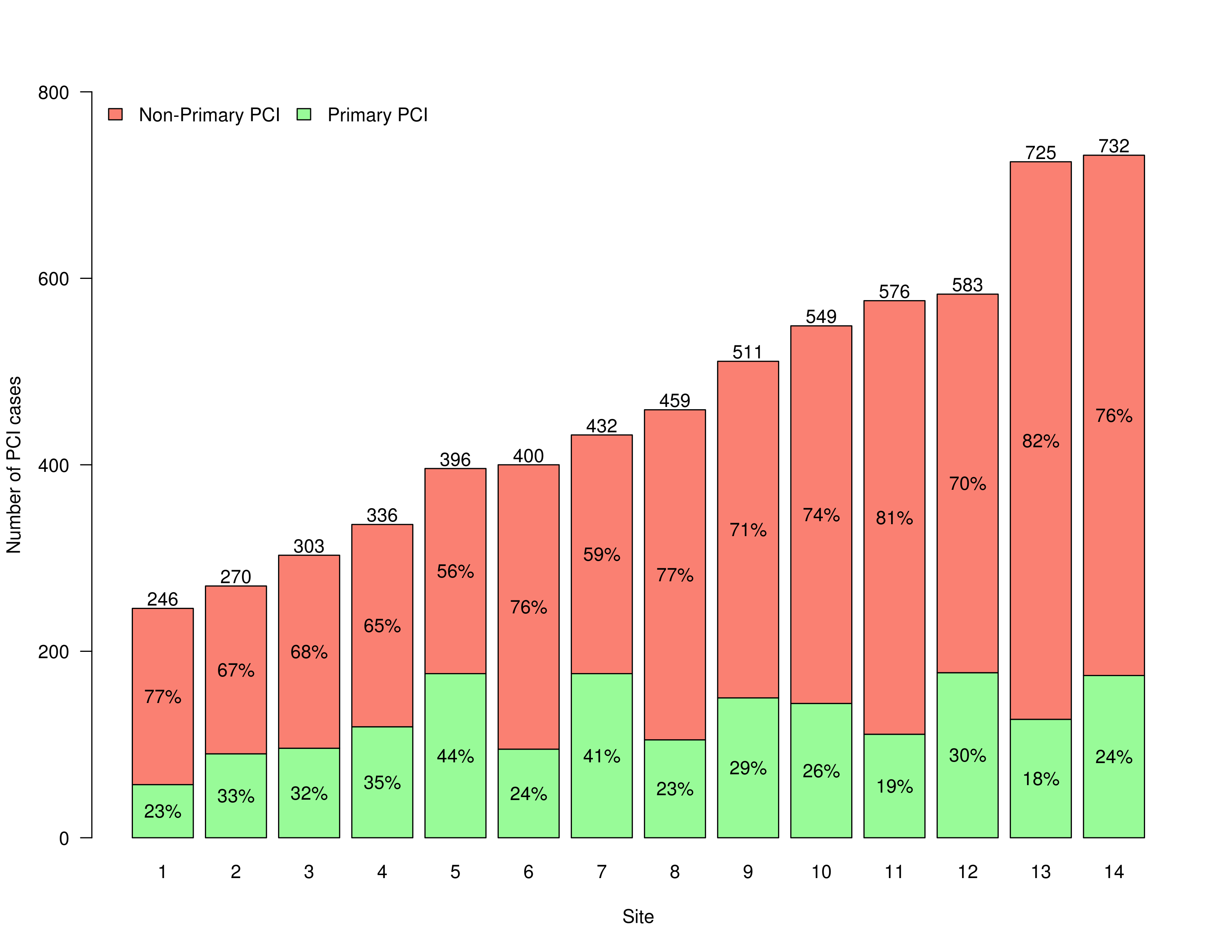

Supplement: S2 Fig — Total PCI volumes over the 2-year study period for each of the 14 non-surgical sites included in the study are shown, with breakdown of both primary and non-primary PCI. (TIFF) [file pone.0238048.s008.tiff]
