# Supplementary material for: Trends and outcomes of non-primary PCI at sites without cardiac surgery on-site: The early Michigan experience
Source: PLoS One. 2020 Aug 26;15(8):e0238048. doi: 10.1371/journal.pone.0238048 (PMC7449474; doi:10.1371/journal.pone.0238048)
Supplement: Attachment — Submitted filename: Response to Reviewers.docx [file pone.0238048.s009.docx]

**Response to Reviewers:**

**1. Please ensure that your manuscript meets PLOS ONE's style requirements, including those for file naming.**

All of the requested changes have been made to fit the guidelines outlined by the journal.

**2. Please refer to the specific statistical analyses performed as well as any post-hoc corrections to correct for multiple comparisons. If these were not performed please justify the reasons.**

Statistical methods used, along with software version used for our analyses are described under our methods, sub-sectional “statistical analysis.” In accordance with journal guidelines, p-values have been adjusted to stay within recommended decimal places. Additionally, we have added a post-hoc analysis of secondary composite endpoint including in-hospital mortality, CVA, stent thrombosis, and target lesion revascularization. We have added an adjusted p-value for our initial MACE endpoint accounting for multiple comparisons using the Bonferroni method.

**3. In the ethics statement in the manuscript and in the online submission form, please provide additional information about the patient records used in your retrospective study.**

**Specifically, please ensure that you have discussed whether all data were fully anonymized before you accessed them and/or whether the IRB or ethics committee waived the requirement for informed consent.**

As described in our methods section (under “Data Source”), our protocol received approval or waiver by the local IRB at each institution as the registry is part of a quality initiative process. Specifically, no unique patient identifiers are collected and informed consent was waived. All patient data points were derived from a HIPAA-compliant database.

**4.We noticed you have some minor occurrence of overlapping text with the following previous publication(s), which needs to be addressed:** [https://www.sciencedirect.com/science/article/abs/pii/S0002870317303162?via%3Dihub](https://www.sciencedirect.com/science/article/abs/pii/S0002870317303162?via%3Dihub)

**In your revision ensure you cite all your sources (including your own works), and quote or rephrase any duplicated text outside the methods section. Further consideration is dependent on these concerns being addressed.**

Thank you for this suggestion. Our prior analysis looked at primary PCI among surgical and non-surgical sites with many similarities, however, the current analysis is quite unique with additional important information. We have made a combination of revisions to the language, along with addition of citations of our prior work and placed certain statements in quotation as well.

**5.We note that you have indicated that data from this study are available upon request. PLOS only allows data to be available upon request if there are legal or ethical restrictions on sharing data publicly. For information on unacceptable data access restrictions, please see** [http://journals.plos.org/plosone/s/data-availability#loc-unacceptable-data-access-restrictions](http://journals.plos.org/plosone/s/data-availability#loc-unacceptable-data-access-restrictions).

**In your revised cover letter, please address the following prompts:**

**a) If there are ethical or legal restrictions on sharing a de-identified data set, please explain them in detail (e.g., data contain potentially identifying or sensitive patient information) and who has imposed them (e.g., an ethics committee). Please also provide contact information for a data access committee, ethics committee, or other institutional body to which data requests may be sent.**

The authors are unable to share the raw data due to the nature of contractual obligations with participating hospitals; the analysis code is available on request from Milan Seth ([mcseth@med.umich.edu](mailto:mcseth@med.umich.edu)). All relevant data are within the paper and its supporting information files.

**6. Thank you for stating the following in the Competing Interests section: 'Competing interests: Hitinder S. Gurm receives research funding from BCBSM and the National Institutes of Health and is a consultant for Osprey Medical. The authors have declared that no competing interests exist. a. Please confirm that this does not alter your adherence to all PLOS ONE policies on sharing data and materials, by including the following statement: "This does not alter our adherence to PLOS ONE policies on sharing data and materials.” (as detailed online in our guide for authors http://journals.plos.org/plosone/s/competing-interests).  If there are restrictions on sharing of data and/or materials, please state these. Please note that we cannot proceed with consideration of your article until this information has been declared. b. Please include your updated Competing Interests statement in your cover letter; we will change the online submission form on your behalf.**

We have updated, confirmed and included this information in our updated cover letter as above

**7. PLOS requires an ORCID iD for the corresponding author in Editorial Manager on papers submitted after December 6th, 2016. Please ensure that you have an ORCID iD and that it is validated in Editorial Manager. To do this, go to ‘Update my Information’ (in the upper left-hand corner of the main menu), and click on the Fetch/Validate link next to the ORCID field. This will take you to the ORCID site and allow you to create a new iD or authenticate a pre-existing iD in Editorial Manager. Please see the following video for instructions on linking an ORCID iD to your Editorial Manager account: [https://www.youtube.com/watch?v=_xcclfuvtxQ](https://www.youtube.com/watch?v=_xcclfuvtxQ)**
The ORCID ID of Dr. Gurm is 0000-0002-1646-0218

**3. Have the authors made all data underlying the findings in their manuscript fully available?**
As previously stated, the authors are unable to share the raw data due to the nature of contractual obligations with participating hospitals; the analysis code is available on request from Milan Seth ([mcseth@med.umich.edu](mailto:mcseth@med.umich.edu)). All relevant data are within the paper and its supporting information files.

**Reviewer Comments:**

**Reviewer #1: 1. Thanks for your research about the safety of elective PCI. The term "elective" is unusual as you have many NSTEMI and UA in your registry that require urgent or semi urgent PCI. For the other cases, I haven't found if the ischemia was previously documented and, when documented, the severity of ischemia. Despite your propensity matched score, we observe more prior MI, more CVD, more previous HF, more left main disease in the group of patients who benefit from a PCI in a site with a surgical department.**

We would like to thank the reviewer for their detailed comments and insight into our manuscript. For more accurate representation and to reduce confusion of readers, we have changed “Elective” to “Non-primary” PCI throughout the manuscript. We have additionally added the number of vessels intervened upon in Table 1, so that differences between groups could be displayed. While we acknowledge the differences in prior cardiac history between groups, as the reviewer stated, these differences did not affect the composite outcomes, or important secondary clinical and quality outcomes. The goal of this manuscript was to demonstrate how this practice pattern manifests in real-world practice outside the limitations of randomized studies or meta-analyses.

**2. Femoral access remains high 52% and maybe discussed**

While practice patterns may be changing with the implementation of radial access, we believe this percentage remains in line with current practice patterns. In fact, several studies have shown significantly higher use of femoral access (e.g. Shamim S et al. Am J Cardiol. 2017;119(12): 1937-41), but growing trends towards increased use of radial access have emerged in the last couple decades. As such, we don’t consider the percentage of femoral access this study to be out of the ordinary with the current US practice.

**3. The same for the high use of Ticagrelor, high probably for NSTEMI. Therefore the term "elective" may be changed.**

We agree with the reviewer. We have changed the term “elective” to “non-primary.”

**4. These data confirm several previous data. They are reassuring for your state, but what are the new informations for the readers?**

While our analysis confirms data of previous studies, unlike randomized controlled trials, which are highly regulated with regards to treatment approaches, and meta-analyses that are limited by variable sample sizes, selection of included studies, and heterogeneity of methodology and data analysis, our study is reflective of real-world practices. Furthermore, we advocate for the use of rigorous quality oversight programs (e.g. BMC2) that use robust systems of data collection, auditing and quality improvement to ensure safe and optimal expansion of PCI services. We have highlighted this in the discussion section.

**5. Multivessel disease and poylarterial disease are not reported and may be on interest in the discussion.**

Thank you for this comment. We agree this is an important piece of information and have included the data of # vessels intervened upon in Table 1.

**6. What's your definition of "high risk" patients and low risk patients?**
“High risk” patients are defined under our statistical analysis section and include patients who undergo interventions on the left main coronary artery, chronic total occlusions, bifurcation lesions, patients with a pre-procedural ejection fraction less than 30%, or the use of atherectomy devices. No specific definition was used for “low risk.”

**Reviewer #2:**
**The authors have undertaken an analysis of the BMC-2 registry studying in hospital and post discharge outcomes in patients undergoing PCI stratified by whether the PCI was undertaken in a surgical vs. non-surgical centre. The authors report no differences in in-hospital outcomes, 90 day readmissions or cost of procedure following PSM analysis in the whole population as well as a high risk subgroup. The analysis adds to the large body of literature published in this arena already, but presents additional data including the readmission data, and a subgroup analysis around a higher risk cohort. The manuscript is well written. I have number of recommendations:**

We would like to thank the reviewer for their detailed review of our manuscript and appreciate their comments and suggestions.

**1) Throughout the manuscript the authors refer to the cohort as an elective PCI cohort. This is misleading, UA and NSTEMI cases for the majority of PCI procedures in this analysis. They should probably rename their cohort as a nonPPCI or ACS / elective cohort.**

We agree with the reviewer. We have changed the term “elective” to “non-primary.”

**2) Their choice of MACE is unusual. they have included CIN (which contributes the majority of outcome events). This is inappropriate. MACCE should probably be in hospital mortality, CVA, AMI/ Stent thrombosis and perhaps re-intervention. NACE could include major bleeding.**

Thank you for this comment. Our a priori hypothesis and statistical plan was pre-defined and including in-hospital mortality, contrast-induced nephropathy (CIN), stroke, National Cardiovascular Data Registry (NCDR)–defined bleeding, and major bleeding defined as ≥5 g/dL drop in hemoglobin as we felt these were important quality outcomes. In response to this review, we have additionally added a secondary composite endpoint composed of in-hospital mortality, CVA/stroke, stent thrombosis, and target lesion revascularization. There was no statistical or clinically significant difference between the two groups using this end point.

**3) The 90 days data is limited to only 10% of population that they could match to medicare claims database. I think that this should be more overt in the abstract, as it is slightly misleading otherwise. The limitations should be re-emphasized in the limitations section of discussion**

We agree the data on this sub-analysis is limited, but still provides important insight. We have clarified in both our abstract and manuscript that this data is derived from a “small” subset of patients and have commented on this in our limitations as well.

**4) It would be good to have a figure showing centre volume in the individual centres, as I think that is useful for the reader. Where there is a lot of data around centre-volume outcomes, do the authors think it is a good idea that activity is redistributed from higher to lower volume centres? (authors report redistribution of activity and discuss it in the context of patients having PCI more locally)**

We agree that this information would be helpful. In response, we have included an additional supplemental figure (S2 Fig), which displays total PCI volume over the study period at each of the 14 non-surgical sites so that the readers may be aware of this information. While redistribution of activity may not necessarily be a good idea overall, we do see the benefits of accommodating patients to remain closer to their residence and family, and within the same integrated or affiliated healthcare systems. Furthermore, we did not see significant differences in outcomes as a result of this redistribution of activity.

**5) Urgent / emergent surgery-was this due to complications? perhaps if data available re indication of this surgery would be very interesting as very little of this type of data.**
While this is an interesting question, unfortunately the database used for this study does not specify the exact indication for the CABG.
